# Supplementary material for: Prophylactic Medication during Radioembolization in Metastatic Liver Disease: Is It Really Necessary? A Retrospective Cohort Study and Systematic Review of the Literature
Source: Diagnostics (Basel). 2023 Dec 12;13(24):3652. doi: 10.3390/diagnostics13243652 (PMC10742749; doi:10.3390/diagnostics13243652)
Supplement: Supplementary file 1 [file diagnostics-13-03652-s001.zip › diagnostics-2711565-supplementary.pdf]

Supplementary Materials

Supplementary Table S1: Hepatotoxicity score*

**Grade 0**	No liver toxicity (i.e. no CTCAE toxicity grade changes over baseline).
**Grade 1**	Minor liver toxicity, limited to increased AST, ALT, ALP and/or GGT levels (all not exceeding newly developed grade 1 CTCAE toxicity).
**Grade 2**	Moderate liver toxicity, with a self-limiting course. No medical intervention necessary.
**Grade 3**	REILD, managable with non-invasive treatments such as diuretics, ursodeoxycholic acid and steroids.
**Grade 4**	REILD necessitating invasive medical treatment such as paracentesis, transfusions, haemodialysis or a transjugular intrahepatic portosystemic shunt (TIPS).
**Grade 5**	Fatal REILD.

*Grading according to Braat et al. [17]

Abbreviations: ALP = alkaline phosphatase, ALT = alanine aminotransferase, AST = aspartate aminotransferase, GGT = gamma-glutamyl transferase, CTCAE = Common Terminology Criteria for Adverse Events, REILD = radioembolization-induced liver disease

Supplementary Table S2: Literature search strategy

**Main search string**
("radioembolic"[All Fields] OR "radioembolisation"[All Fields] OR "radioembolization"[All Fields] OR radio-emboli* [All Fields] OR "SIRT"[All fields] OR "selective internal radiation therapy"[All Fields] OR "TARE" [All Fields] OR "transarterial radioembolization"[All Fields])<br>**AND**<br>(("liver"[MeSH Terms] OR "liver"[All Fields] OR neoplasm metastasis[MeSH Terms] OR liver neoplasm[MeSH Terms]))<br>**AND**<br>("octreotid"[All Fields] OR "octreotide"[MeSH Terms] OR "octreotide"[All Fields] OR "somatostatin"[All Fields] OR SSA[All Fields] OR "Anti-Bacterial Agents"[Mesh] OR "Antibiotic Prophylaxis"[Mesh] OR antibioti* [All Fields] OR anti-emetic [All Fields] OR prophylaxis [All Fields] OR analgesics [All Fields] OR steroids[All Fields])

Supplementary data on the literature review of PPI/H2 antagonists

Concerning other PPM (proton pump inhibitors (PPI), H2-antagonists and antibiotics), efficacy is even more questionable and proper evidence is lacking. Lim et al. advised H2-antagonists, because of gastroscopy proven gastrointestinal ulceration in 8% (4 patients, of which 3 biopsy proven) [20]. The applicability of H2-antagonists or PPI in contemporary practice is debatable since most centers perform improved pre-treatment work-up with perprocedural conebeam-CT and ^{99m}Tc-MAA SPECT/CT to prevent extrahepatic deposition of activity. More recent studies looking at radiation absorbed doses to the stomach after left lobe treatments showed negligible absorbed doses (i.e. 13±9 Gy) and low rates of GI-ulceration (<3%) [24–27]. Although questionable, in absence of a comparator group (i.e. without any form of PPM) no definitive judgment can be made on the efficacy of H2-antagonists or PPI.
